# Using resting-state DMN effective connectivity to characterize the neurofunctional architecture of empathy

**DOI:** 10.1038/s41598-019-38801-6

**Published:** 2019-02-22

**Authors:** Sofia Esménio, José M. Soares, P. Oliveira-Silva, Peter Zeidman, Adeel Razi, Óscar F. Gonçalves, Karl Friston, Joana Coutinho

**Affiliations:** 1 Neuropsychophysiology Lab, Psychology School, Minho University, Campus Gualtar, Braga, Portugal; 2 Life and Health Sciences Research Institute (ICVS), School of Health Sciences, University of Minho, Campus Gualtar,, Braga, Portugal; 3 ICVS/3B’s – PT Government Associate Laboratory, Braga/Guimarães, Portugal; 4 Clinical Academic Center, Braga, Portugal; 5 Faculty of Education and Psychology, Catholic University of Portugal, Porto, Portugal; 6 Applied Psychology Bouvé College of Health Sciences Northeastern University Harvard Medical School, Boston, USA; 7 Wellcome Trust Centre for Neuroimaging, University College London, London, UK

## Abstract

Neuroimaging studies in social neuroscience have largely relied on functional connectivity (FC) methods to characterize the functional integration between different brain regions. However, these methods have limited utility in social-cognitive studies that aim to understand the directed information flow among brain areas that underlies complex psychological processes. In this study we combined functional and effective connectivity approaches to characterize the functional integration within the Default Mode Network (DMN) and its role in self-perceived empathy. Forty-two participants underwent a resting state fMRI scan and completed a questionnaire of dyadic empathy. Independent Component Analysis (ICA) showed that higher empathy scores were associated with an increased contribution of the medial prefrontal cortex (mPFC) to the DMN spatial mode. Dynamic causal modelling (DCM) combined with Canonical Variance Analysis (CVA) revealed that this association was mediated indirectly by the posterior cingulate cortex (PCC) via the right inferior parietal lobule (IPL). More specifically, in participants with higher scores in empathy, the PCC had a greater effect on bilateral IPL and the right IPL had a greater influence on mPFC. These results highlight the importance of using analytic approaches that address directed and hierarchical connectivity within networks, when studying complex psychological phenomena, such as empathy.

## Introduction

Neuroimaging studies have made important contributions to our understanding of the neural basis of several psychological processes (e.g.^1–3^). A variety of brain connectivity methods have been used to study the brain networks underlying these psychological processes, using functional magnetic resonance imaging (fMRI) data (see the comprehensive review in^4^. Neuroimaging research in cognitive neuroscience, sometimes rely on functional connectivity (FC) methods (e.g., seed based analysis and independent component analysis - ICA) to describe the relationship between different brain regions^5–7^.

However, the utility of functional connectivity methods is limited in social-cognitive studies, where the goal is to infer specific mental processes (e.g., theory of mind) from observed patterns of regional responses (e.g., in the temporal parietal junction and medial prefrontal areas). This inference is complicated by the fact that a single brain region is often recruited by a wide range of psychological processes and a given cognitive process may recruit more than one brain region^8–10^.

In fact, previous empirical evidence suggests that the more complex a given psychological process is, the more likely it is to engage the cooperation of several brain regions^9,11–13^. This is true for complex psychological functions such as human empathy that comprises several processes; ranging from the ability to feel what the other person is feeling to the capacity to understand a given situation from the perspective of another person in order to anticipate his or her actions^14^. Due to the complexity of the construct of empathy the debate around its definition has been considerable, with some authors defining it in emotional terms^15,16^ and others defining it in cognitive terms^17,18^. One approach often used in the literature^19–22^ to deal with this controversy is to adopt a multidimensional definition of empathy which includes both basic affective processes (e.g., emotional contagious and the ability to share affective states evoked by another individual), as well as more conceptual and cognitive dimensions (e.g., the ability to identify and understand the mental states of others). Similarly, in our work we define empathy as our ability to share, react to and understand the emotions of others. As we will see later, this was reflected in the empathy measure that we selected for this study, which assesses both emotional and cognitive dimensions of self-perceived empathy.

More specifically, in this study we focused on empathy expressed towards the romantic partner which is referred to as dyadic empathy^23^. Contrarily to general empathy that corresponds to the empathic tendencies in a general social context, not specific to a particular relationship, we were interested in looking at empathy expressed within the romantic relationship. Among the various dyadic interactions in which empathy has been studied, romantic relationships becomes an especially interesting context of study, since it is critically depending on feelings of compassion, support and empathic validation^24^. In fact, by allowing one partner to share and understand the other internal states, empathy is essential for stable and satisfactory couples relationships^25,26^.

Extensive literature on empathy and the brain has consistently demonstrated that different neurobiological systems are involved in the various dimensions of empathy (see^12^ for a review). For example, we know that experiencing another person’s feelings recruits emotional brain circuits comprised by anterior insula, parahippocampal gyrus, amygdala and anterior cingulate cortex^12,27^, whereas our ability to cognitively understand other’s feelings and thoughts recruits the medial prefrontal cortex, temporal parietal junction and posterior cingulate cortex^27,28^.

The latter regions belong to the Default Mode Network (DMN) which is one of the better known resting state networks that comprises four key anatomical regions: the mPFC, the PCC and the left and right inferior parietal lobule (lIPL and rIPL)^29–32^. This network whose regions seem to be recruited when we think about mental states, either ours and those of others, has been consistently associated with social functions in general^33,34^ and empathic abilities not only in humans^2,33,35^ but also in rodents and primates. In fact, the DMN is of particular interest for the study of the neural basis of empathy, because it has been consistently associated with several psychological processes involved in it. The DMN has not only been associated with the understanding of another’s pain through the inner representation of their affective states^2^, but also with the ability to self-regulate the emotional arousal evoked by that vicarious experience in order to maintain an adequate distinction between our own and other’s psychological states.

Despite the well documented role of the DMN for empathic processes, less is known about the dynamic interplay between its nodes and its importance for empathic processes. We consider that this is due to the fact that so far most studies looking at the involvement of the DMN in empathy, used traditional functional connectivity approaches.

In fact, as mentioned before, empathy is a very complex phenomena and when analyzing the brain responses underlying such high-level psychological processes, there is a need for analytic approaches that characterize the cortical hierarchies and directed causal relationships among brain areas within a network.

In contrast with functional connectivity (FC) approaches that are usually inferred on the basis of correlations among measurements of neuronal activity (i.e. which voxels display similar BOLD signal fluctuations over time), effective connectivity (EC) is defined as the directed influence that one brain region exerts over another^36,37^, supplementing FC in a complementary manner. While FC detects consistent spatiotemporal relationships between different brain regions, EC analyses considers how the information flow through these brain regions^37,38^ providing insight into how the brain activity comes about.

In particular, dynamic causal modelling (DCM) infers effective connectivity (EC) between neuronal populations by combining dynamic models of neuronal states and detailed biophysical hemodynamic models^39^. DCM supplements a forward model – of how cortical regions interact – with a hemodynamic model that transforms neuronal activity into measured response (e.g., blood oxygen level response)^40–42^. DCM has been used extensively to study task-related brain responses^43–47^; however, recent DCM methods now also support inference on connectivity during resting-state – by modelling endogenous neural fluctuations^40,41^: this is referred to as spectral DCM. DCM of effective connectivity at rest has been applied to clinical phenomena like melancholia^48^; schizophrenia (e.g.^49,50^) and smoking addiction^51^.

The use of DCM in this context may also be beneficial to the understanding of normal psychological processes, such as empathy, that entail distributed processing over large-scale networks. Thus, in this study we used spectral DCM^40,41^, to analyze the effective connectivity within the DMN – and to quantify how the functional architecture of this brain network relates to self-perceived empathy. Our reasoning was that by understanding how the information flow among DMN regions is related with differences in empathy abilities, we will better understand the role of each node in empathy (e.g.^52^). For this purpose, we used both a functional (by means of an ICA analysis) and effective connectivity (by means of a DCM analysis) to analyze the relationship between the DMN functional architecture and self-perceived empathy. In summary, our hypothesis was that variations in the self-perceived empathy would be underwritten by systematic differences in effective connectivity among the nodes of the default mode network – and that these differences in directed coupling mediate differences in functional connectivity.

## Results

### Functional connectivity results

**Figure 1:**
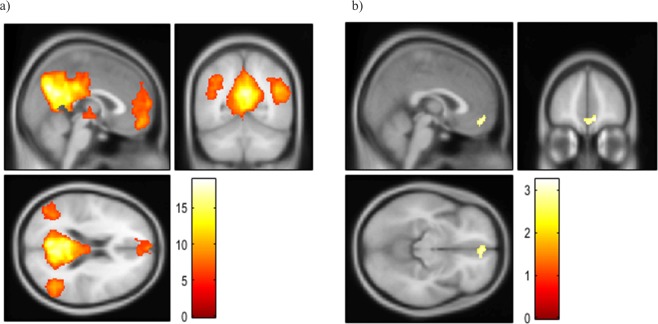
(**a**) Consistent DMN expression identified in the resting state over subjects. (**b**) Correlation between IRIC total score and DMN functional connectivity.

The spatial modes (i.e., independent components) of FC in the DMN during resting state were identified at the group level and four main regions were observed; namely, the PCC and precuneus (PCu) (x = 2; y = −60; z = 30; Z > 8, 9767 voxels), the bilateral IPLs (x = −40; y = −70; z = 40; Z > 8, 2339 voxels and x = 44; y = −64; z = 32; Z = 7.74, 1746 voxels) and the mPFC (x = −6; y = 52; z = 8; Z = 7.60, 1941 voxels) (Fig. 1a). At rest, increased IRIC total score was associated with an increased expression of the DMN in the medial prefrontal cortex (mPFC), primarily in the frontal pole (peak MNI x = 10; y = 50; z = −10; Z = 2.92, 117 voxels) (Fig. 1b).

### Effective connectivity results

**Figure 2:**
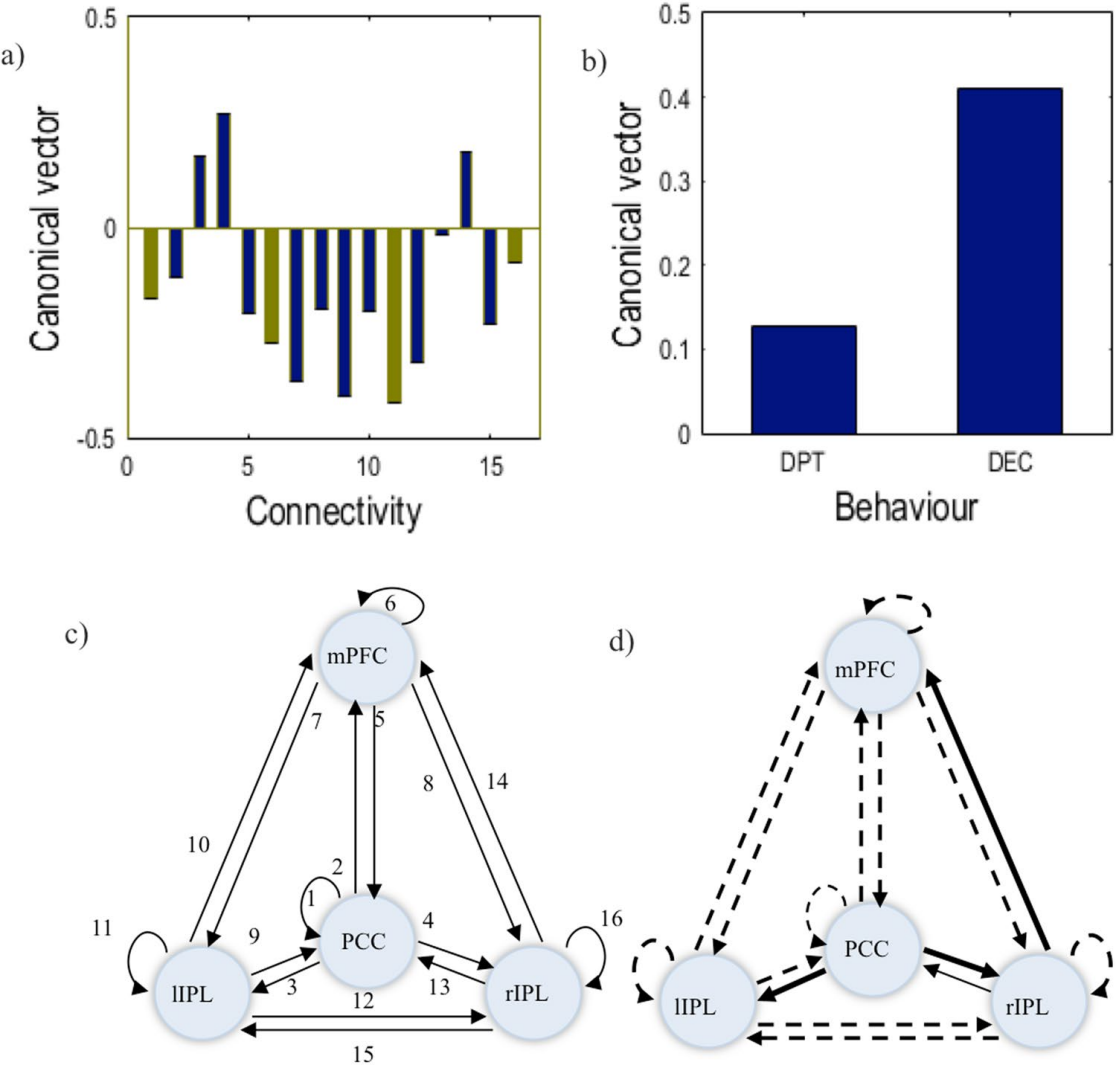
(**a**) Canonical Vector containing weight (contribution) assigned by CVA to each DCM effective connectivity parameter. Blue connections correspond to extrinsic connections and green to intrinsic connections. (**b**) Canonical Vector containing weight (contribution) assigned by CVA to each empathy scale score: perspective taking (PT) and empathic concern (EC). (**c**) Showing the index number of each effective connection in **a**. (**d**) Weight of each connection, where bold lines represent an increase and dashed lines a decrease with empathy.

Canonical variate analysis of the multivariate relationship between DCM effective connectivity estimates and dyadic empathy scores showed a significant canonical correlation for the first canonical vector (p = 0.01598, chi-squared statistic (*X*^2^) = 33.1996). This vector showed that higher IRIC total scores (M = 40.36, SD = 4.50) were associated with an overall decrease in connectivity between regions and regional self-inhibition (Fig. 2a).

To understand the importance of these results, an important distinction needs to be made between extrinsic or direct connections (i.e., connections that reflect the EC between regions - represented in blue in Fig. 2a) and intrinsic or self-inhibition connections (i.e. connections that reflect how susceptible a node is to extrinsic afferent - represented in green in Fig. 2a). In terms of extrinsic connections, there was a general decrease of the influence of all DMN nodes on each other, except from: PCC to rIPL (4); rIPL to PCC (13); PCC to lIPL (3) and rIPLto mPFC (14) (Fig. 2a,d). In other words, there was a general decrease in effective connectivity accompanied with a selective increase in the influence of PCC on bilateral IPL, and a right hemisphere increase in the influence of IPL on the medial prefrontal cortex.

Regarding the intrinsic connections, the results show lower self- inhibition in all nodes with higher empathy scores; (Fig. 2a,d) specially in PCC (6) and lIPL (11) (Fig. 2d). In this case, a smaller self-inhibition in a node represents an increase in sensitivity to extrinsic afferents, i.e. the node becomes more excitable, taking less time to respond to the influence of other nodes.

In addition, this canonical vector also highlights that empathic concern in comparison with perspective taking, has a greater contribution to the canonical correlation; i.e., a greater predictive validity in terms of EC within the DMN (Fig. 2b).

## Discussion

The current study examined the extent to which individual differences in self-reports of dyadic empathy were reflected in functional integration within the DMN. Differences in DMN connectivity were addressed both in terms of functional and effective connectivity among different brain areas, by means of ICA and DCM respectively. Our results showed significant correlations between DMN’s functional architecture and self-reported dyadic empathy.

In terms of functional connectivity, ICA showed that higher scores in dyadic empathy were associated with a significant increase in the expression of the mPFC; more specifically, in the frontal pole. The mPFC has been linked with our capacity to reflect on the mental state of others^53,54^, and the frontal pole has been shown to play a crucial role in the functional integration between cognition and emotion; particularly, in the case of adaptive empathic responses^19,55,56^. In line with our results, previous literature using ICA showed that when compared to a medium-empathy group, low-empathizers display lower FC of the mPFC within the DMN^57^. This evidence suggests a relationship between empathy and the functional connectivity of the mPFC within the DMN.

Between-subject differences in functional connectivity underlying this result were clarified, mechanistically, in terms of differences in effective connectivity involving not just afferents to the medial prefrontal cortex but other key differences throughout the DMN.

In terms of directed coupling within the DMN, spectral DCM^39–41^ showed a clear and consistent pattern. Increase in empathy scores was associated with a general decrease in EC between DMN nodes that was accompanied with a universal disinhibition in all nodes. In other words, not only did nodes became more self-sufficient but, simultaneously, showed a critical slowing with decreased self-inhibition. This effect was accompanied by an increase in PCC’s influence on bilateral inferior parietal cortices. Essentially, as self-perceived empathy scores increases, the PCC seems to dominate interactions within the DMN. These results are consistent with previous evidence that speaks to the PCC’s role as a connector node for the functional integration within and between brain networks^58–60^.

It is interesting that the particular connections from the PCC that increased with empathy over subjects, were in the descending direction (i.e., they were PCC efferents as opposed to ascending afferents). This may speak to the fundamental role of top-down predictions about one’s own bodily state when trying to infer the intentions of others^61^; particularly in the context of predictive coding formulations of empathy^62–64^. Additionally, we found that the PCC exerted an excitatory coupling on both IPLs that propagated vicariously to the mPFC, via the rIPL. In other words, participants with higher self-perceived empathy presented with a lateralization in the DMN, with the right IPL acting as the mediator between PCC and mPFC.

This lateralization – through the rIPL – within the DMN is consistent with lesion studies which suggest that empathic abilities are compromised in both patients with prefrontal lesions and patients with lesions on the right parietal cortex^65^. In fact, when lesions involved the left parietal cortex, patients showed no impairment on empathic ability. This highlights the specific contribution of the right IPL – and mPFC – in empathic processes.

Furthermore, previous research assessing the functional integration in the DMN showed a clear rightward asymmetry of extrinsic connectivity^66^. In addition, this study showed that the right IPL sends information both to the mPFC and the PCC, and the mPFC sends information to the PCC. Therefore, it seems that the higher self-perceived empathy is associated with a decrease in the communication between the mPFC and the PCC and with an inversion of the information flow between rIPL and PCC.

In summary, in agreement with the consistent relationship found in the literature between the DMN and human empathy^2,33,35^, our results show a pattern of intrinsic connectivity changes in this network associated with differences in dyadic empathy measures. Furthermore, systematic differences in information flow – among the DMN regions – showed that in individuals with higher dyadic empathy, the PCC seems to assume a central function within the DMN, by influencing, directly, the bilateral parietal regions and, indirectly, the mPFC through the right IPL. Although in the functional connectivity analysis the mPFC was the node associated with higher self-perceived empathy, the results from the DCM analysis suggest that this increase results from a shift in coupling between PCC and rIPL. These results highlight the importance of applying effective connectivity analysis, such as DCM, when trying to characterize more complex psychological phenomena, such as empathy.

Finally, the results of the CVA analysis^67^ include the relative contribution of each of the IRIC scales (i.e., empathic concern and perspective taking) to the previously mentioned differences found in DMN connectivity. Results showed that while both subscales contributed positively to the observed changes in connectivity within the DMN, the empathic concern subscale presented a significantly higher contribution to those changes.

In previous studies^34^, reports that DMN acts as a physiological “baseline” of the human brain that is linked to our predisposition for social cognition. Similarly, our results suggest the DMN as a network that is linked to more ‘affective–perceptual’ forms of empathy, hence empathic concern.

Although our results are compelling, the current study is not free from the limitations inherent to all the resting state studies; such as the difficulty with controlling behavior related confounds such as sleep and the unconstrained nature of the “task”. In fact, test–retest reliability of resting state connectivity measures typically range between moderate to good even with optimal processing^68,69^, which may be due to the absence of cognitive constraints. Future studies could try to monitor the cognitive processes at rest by using some measures such as mind wandering questionnaires – or indeed a carefully designed empathy task to look at condition-specific changes in effective connectivity. Furthermore, it would be interesting to apply advanced (second level) data analysis method in combination with DCM (e.g. parametric empirical Bayes – PEB), to assess the specific contribution of each self-report subscale (e.g. empathic concern) on each specific connection (e.g. PCC to rIPL). Finally, it could be interesting to examine the existence of similar connectivity patterns in other resting state networks that include regions associated with empathy response, namely the Salience Network that includes both the anterior insula & dorsal ACC^70,71^.

## Methods

### Participants

Forty-two individuals, comprising 21 heterosexual couples that reported being in committed monogamous romantic relationship for at least one year, participated in this study. Participants’ ages ranged from 23 to 40 years old (M = 31.17, SD = 4.748; for males: M = 32.13, SD = 4.893, for females: M = 30.22, SD = 4.502). Prior to any procedure, all participants were screened on the telephone to assess inclusion and exclusion criteria. Exclusion criteria were as follows: (1) the presence of any dementia and/or diagnosed neuropsychiatric and/or neurodegenerative disorder; (2) dependency or abuse in the past year of alcohol and/or drugs; (3) incapacity and/or inability to attend the MRI session (e.g., metallic implants; pregnancy); (4) age bellow 20 or above 50 years. All participants were Caucasian and right-handed.

The study goals and procedures were explained and all the participants gave informed written consent. All the procedures of this study were approved by the Institutional Review Board of University of Minho and the study was conducted in accordance with the principles expressed in the Declaration of Helsinki.

### Empathy Measures

To assess dyadic empathy, participants completed the Portuguese version of the Interpersonal Reactivity Index for Couples (IRIC)^24,72^. The IRIC is a modified version of the Interpersonal Reactivity Index (IRI) that assesses cognitive and emotional empathy in the context of intimate relationships. It is composed by 13-items that assess empathy toward the partner specifically (e.g.: *“I* sometimes try to understand my partner better by imagining how things look from his/her perspective”). The cognitive aspects of empathy are measured by the Dyadic Perspective-Taking (DPT) subscale, which evaluates individuals’ ability to adopt the psychological point of view of their partner. The emotional aspects are measured by the Dyadic Empathic Concern (DEC) subscale, which evaluates individuals’ feelings of concern for their partner. Each questionnaire item is measured on a 5-point Likert-type scale, with responses ranging from: (0) “Does not describe me well” to (4) “Describes me very well”. Higher scores in a scale represent greater perspective-taking or empathic concern. The IRIC total score can vary between 0 and 52, with a higher score indicating higher perceived empathy within the couple’s relationship. The score of dyadic perspective taking can vary between 0 and 24 and the score of dyadic empathic concern can vary between 0 and 28. The Portuguese version of IRIC showed adequate internal consistency values (Cronbach’s α = 0,82) and good levels of external validity with a dyadic adjustment scale (DAS)^72^.

The administration of the IRIC occurred before the neuroimaging acquisition and took place in the lab using a paper and pencil version. The IRIC was administered anonymously and independently to each element of the couple, that is, each spouse completed the questionnaire individually and was asked not to exchange impressions on items or their answers with the partner. A member of the research team was present in the room to ensure the participants had no doubts about the IRIC. In order to control for social desirability effects and ensure the total confidentiality of the answers a code was assigned to each participant and to his/her respective questionnaire, which, after completed, was introduced in a sealed envelope. The values obtained in our sample for the scores are comparable to those reported with other samples^70^ :DPT, M = 16.37, SD = 3,83 and DEC, M = 23,72, SD = 2.951.

### MRI acquisitions

MRI data were acquired with a clinically approved 3Tesla MRI scanner (Siemens Magnetom Tim Trio, Erlangen, Germany). A 7-min resting-state functional acquisition (210 volumes) was obtained for each participant using a sensitive echo-planar imaging (EPI) BOLD sequence with the following imaging parameters: repetition time (TR) = 2000 ms; echo time (TE) = 29 ms; flip angle = 90°; field of view (FoV) = 1554 mm; matrix size = 64 × 64; pixel size = 3 × 3 mm^2^, slice thickness = 3 mm and 39 slices. Additionally, one structural scan (192 sagittal slices, repetition time (TR) = 2000 ms; echo time (TE) = 2.33 s, flip angle = 7°, slice thickness = 0.8 mm, slice gap = 0 mm, pixel size = 0.8 × 0.8 mm^2^, field of view (FoV) = 256 mm) was also acquired. Participants were instructed to keep their eyes closed and to remain awake but relaxed, doing nothing during the acquisition and remaining as motionless as possible. None of the participants fell asleep during the acquisition.

### Functional connectivity pre-processing and analysis

Image pre-processing and spatial independent component analysis (ICA) was performed with tools provided with the FMRIB Software Library (FSL v5.09; http://fsl.fmrib.ox.ac.uk/fsl/)^73–75^. Preprocessing steps included: removal of the first five volumes (10s) to ensure signal stabilization and allow the participants to adjust to the scanner noise; slice-timing correction using the first slice as reference; motion correction (using rigid body alignment of each volume to the mean image of the acquisition using MCFLIRT) & motion scrubbing (volumes in which FD > 0.5 and DVARS > 0.5% change in the BOLD signal were “scrubbed,” or removed entirely from the data^76^; non-linear normalization to the MNI standard space (using the structural T1 normalization matrix); regression of motion parameters, mean WM and CSF signals (generated by segmenting each individual’s structural image using FAST, thresholded to ensure 80% tissue type probability); band-pass temporal filtering (0.01–0.08 Hz) and spatial smoothing (8 mm full width at half-maximum Gaussian kernel)^4^. Before any data processing and analysis, all acquisitions were visually inspected to confirm that they were not affected by undue head motion and that participants had no brain lesions. On average, 6 volumes were removed per participants (scrubbing correction) and three participants were excluded due to the presence of head motion of greater than 3 mm in translation or 1.5° in rotation.

Next, the spatial ICA was performed for each participant, but across all participants. Briefly, spatial ICA analysis is a multivariate data-driven approach that uses temporal correlations to compute spatial maps of functionally connected regions, while maximizing the independence between them^77,78^. To perform the spatial ICA, first we used MELODIC to search for common spatial patterns among subjects and automatically estimate the number of independent components to extract. After performing the group ICA, dual regression was applied to recover each subject’s version of the group components. Pearson correlation coefficients were then calculated between the time-series of each pair of components ROIs, resulting in one correlation matrix per subject, which were then transformed to Z-score matrices by the application of Fisher’s transform. The final 21 components identified by ICA were visually inspected, sorted, and associated with the resting state functional networks from^79^. This revealed 11 independent resting state networks with typical spatial patterns of functional connectivity. The component that corresponded to the DMN was identified for the final sample (N = 39). The resulting independent component or spatial mode was used as a summary of functional connectivity (implicit in the ICA).

Our initial analysis comprised a one-sample t-test in SPM12 (Wellcome Department of Cognitive Neurology, London, UK) to identify regions within the default mode that were conserved over subjects. A second analysis was then performed to test for a relationship between dyadic empathy – as assessed by the IRIC and the expression of the DMN. Statistical parametric mapping was computed at the between subject level using subject specific DMN maps as the response variable and IRIC’s total score as the explanatory variable in a general linear model. This model included age and gender as nuisance variables. Results were considered significant at p < 0.05, corrected for multiple comparisons using the Monte Carlo correction. The correction was determined for clusters with a height threshold of p ≤ 0.01 and a size threshold of 116 contiguous voxels. A combination of visual inspection and Anatomical Automatic Labeling atlas (AAL) was used for anatomical labelling^80^.

### Effective connectivity pre-processing and analysis

In a second data analysis stream, preprocessing and subsequent dynamic causal modelling were performed using the Statistical Parametric Mapping software (SPM12; Wellcome Department of Cognitive Neurology, London, UK). The preprocessing steps included: removal of the first five volumes; slice-timing correction; motion correction (re-aligned to the mean image); normalization of the functional acquisition to the MNI standard space through the sequential application of a rigid body transformation and the nonlinear warp resultant of previous nonlinear registration of the structural scan to the MNI T1 template^81^; regression of motion parameters, mean WM and CSF signals (generated by segmenting each individual’s structural image using SPM, thresholded to ensure 80% tissue type probability) and smoothing using an 8 mm full width at half-maximum Gaussian kernel. After processing, images were visually inspected to ensure that they had not any undue head motion and that participants had no brain lesions. As above, three participants were excluded as they exceed a head motion higher than 3 mm (translation) or 1.5° (rotation).

Spectral DCM (spDCM) was used to analyze the resting state fMRI data. In brief, spDCM analysis involves a specification of a plausible network model, which then enables the estimation of the model parameters that quantify effective connectivity and regionally specific haemodynamic variables (Friston *et al*., 2014; Razi *et al*., 2015). Model specification comprised the selection of the regions of interest and definition of the model space in terms of connectivity between regions. Based on the previous literature on DMN neuroanatomy^29–33^, we identified four ROIs as key DMN nodes; including the mPFC, PCC, left and right inferior parietal lobule. The MNI coordinates for these nodes were identified for each participant using previously described ICA analysis. The regions of interest were masked by a (8 mm radius) sphere centered at the participant-specific coordinates. Since there is no previous literature on the relationship between the information flow within DMN and empathy, we adopted and exploratory approach, starting with a fully connected model, i.e. a model in which all the ROIs communicate with each other. A fully connected model was constructed for each subject, containing a total of 16 connectivity parameters: 12 connection between regions and 4 recurrent self-connections (see Fig. 2c). Having specified the full DCM, it was inverted for each participant. Furthermore, Bayesian model reduction (BMR)^82,83^ was used was used to find for each participant the best model (i.e. the relevant connections) to explain the data. BMR selected the model with the highest posterior probability from every possible design nested within the previously defined model (i.e. fully connected model). Afterwards, to ensure convergence, cross spectra density (CSD) data fit was inspected visually. One participant was excluded due to a poor data fit. Finally, to study the relationship between DMN’s DCM effective connectivity estimates and empathy scores, we performed canonical variate analysis (CVA)^67^. In the CVA, participant’s DCM data was combined with their scores in each subscale of IRIC’s questionnaire (i.e. perspective taking and empathic concern scales). This way it was possible to understand the relative influence of each IRIC’s subscale in the changes in DMN’s connectivity. The canonical vectors were considered significant at p < 0.05.
